# The time of day effects of warm temperature on flowering time involve PIF4 and PIF5

**DOI:** 10.1093/jxb/ert487

**Published:** 2014-02-18

**Authors:** Bryan C. Thines, Youngwon Youn, Maritza I. Duarte, Frank G. Harmon

**Affiliations:** ^1^Department of Plant & Microbial Biology, University of California, Berkeley, CA 94720, USA; ^2^Plant Gene Expression Center, USDA-ARS, Albany, CA 94710, USA

**Keywords:** Circadian clock, CONSTANS, flowering, FLOWERING LOCUS T, PHYTOCHROME INTERACTING FACTOR, PIF4, PIF5, temperature response.

## Abstract

Warm temperature promotes flowering in *Arabidopsis thaliana* and this response involves multiple signalling pathways. To understand the temporal dynamics of temperature perception, tests were carried out to determine if there was a daily window of enhanced sensitivity to warm temperature (28 **°**C). Warm temperature applied during daytime, night-time, or continuously elicited earlier flowering, but the effects of each treatment were unequal. Plants exposed to warm night (WN) conditions flowered nearly as early as those in constant warm (CW) conditions, while treatment with warm days (WD) caused later flowering than either WN or CW. Flowering in each condition relied to varying degrees on the activity of *CO* , *FT* , *PIF4* , and *PIF5* , as well as the action of unknown genes. The combination of signalling pathways involved in flowering depended on the time of the temperature cue. WN treatments caused a significant advance in the rhythmic expression waveform of *CO*, which correlated with pronounced up-regulation of *FT* expression, while WD caused limited changes in *CO* expression and no stimulation of *FT* expression. WN- and WD-induced flowering was partially *CO* independent and, unexpectedly, dependent on *PIF4* and *PIF5* . *pif4-2*, *pif5-3*, and *pif4-2 pif5-3* mutants had delayed flowering under all three warm conditions. The double mutant was also late flowering in control conditions. In addition, WN conditions alone imposed selective changes to *PIF4* and *PIF5* expression. Thus, the PIF4 and PIF5 transcription factors promote flowering by at least two means: inducing *FT* expression in WN and acting outside of *FT* by an unknown mechanism in WD.

## Summary

The timing of warm temperature cues dictates the magnitude of the early flowering response in *Arabidopsis thaliana* and the contribution of *CO*, *FT*, *PIF4*, and *PIF5* to promotion of flowering.

## Introduction

Warm temperature is a powerful cue for flowering in many plant species, and flowering in response to this cue appears to act through multiple signalling pathways, including the components of the photoperiod pathway (Heggie and Halliday, 2005; Wigge, 2013). The photoperiod pathway regulates flowering time by measuring daylength, or photoperiod. Two important genes in this pathway are *CONSTANS* (*CO*) and *FLOWERING LOCUS T* (*FT*) (Imaizumi, 2010). In *Arabidopsis thaliana*, CO activates *FT* expression in a light-dependent fashion (Suarez-Lopez *et al.*, 2001; Yanovsky and Kay, 2002). Regulation of *FT* by CO and other transcription factors is a central means to integrate signals from the photoperiod pathway with those of the autonomous, vernalization, and gibberellin flowering time pathways (Turck *et al.*, 2008). FT serves as the florigen molecule that transmits this integrated flowering signal to the shoot apical meristem to trigger flowering (Corbesier *et al.*, 2007; Jaeger and Wigge, 2007; Lin *et al.*, 2007).

*CO* expression is clock driven and phased to the evening (Suarez-Lopez *et al.*, 2001). CO is stable and active in light, while darkness leads to CO inactivation though protein degradation (Valverde *et al.*, 2004). In long days, *CO* expression coincides with light so that CO is available to promote high *FT* expression; on the other hand, *CO* expression is restricted to the dark during short days, which limits CO accumulation and, as a consequence, *FT* is weakly expressed (Suarez-Lopez *et al.*, 2001; Yanovsky and Kay, 2002; Valverde *et al.*, 2004). Rhythmic *CO* expression is partially imposed by CYCLING DOF FACTOR1 (CDF1), which is a transcription factor that represses *CO* during the early part of the day (Sawa *et al.*, 2007). In the afternoon, blue light promotes interaction of *FLAVIN-BINDING KELCH REPEAT F-BOX1* (*FKF1*) and *GIGANTEA* (*GI*). The FKF1–GI complex relieves repression of *CO* by promoting degradation of CDF1 by the 26S proteasome. Blue light-activated FKF1 also stimulates flowering by interacting with CO to retard its degradation (Song *et al.*, 2012). GI also directly promotes *FT* expression through binding to the *FT* promoter (Sawa and Kay, 2011).

The *Arabidopsis* autonomous pathway is also involved in activation of flowering by warm ambient temperatures. FT appears to be the major integrator of the signals from the autonomous pathway. Floral induction relies on *FCA* and *FVE* (Balasubramanian *et al.*, 2006; Blazquez *et al.*, 2003). In addition, *FLOWERING LOCUS C* (*FLC*) and *FLOWERING LOCUS M* (*FLM*) influence the floral transition at 27 °C in a *CO*-independent fashion (Balasubramanian *et al.*, 2006). While it is not clear how warm temperature is perceived, photoreceptors have several roles in temperature response (Halliday and Whitelam, 2003; Halliday *et al.*, 2003; Foreman *et al.*, 2011). Notably, phyB is a repressor of flowering at higher ambient temperatures (Halliday and Whitelam, 2003; Halliday *et al.*, 2003).

A family of basic helix–loop–helix (bHLH) transcription factors called PHYTOCHROME INTERACTING FACTORS (PIFs) regulate a spectrum of plant developmental processes, including inhibition of seed germination (Alabadi *et al.*, 2008; Oh *et al.*, 2004), skotomorphogenesis (Huq and Quail, 2002; Khanna *et al.*, 2003; Leivar *et al.*, 2008b), the shade avoidance response (Lorrain *et al.*, 2008), hypocotyl and petiole growth (Huq and Quail, 2002; Nozue *et al.*, 2007), as well as other developmental processes (Monte *et al.*, 2007). For example, PIF4 regulates growth at elevated temperatures (Koini *et al.*, 2009; Stavang *et al.*, 2009; Foreman *et al.*, 2011; Franklin *et al.*, 2011). Phytochromes control the activity of PIFs in a light-dependent manner (Castillon *et al.*, 2007; Monte *et al.*, 2007; Leivar and Quail, 2011). phyB physically associates with PIFs in red light. The PIF and phyB proteins in this complex are ubiquitylated and degraded by what appears to be independent mechanisms (Castillon *et al.*, 2007; Monte *et al.*, 2007; Henriques *et al.*, 2009; Jang *et al.*, 2010). The antagonistic relationship of PIFs and phytochromes is apparent in mutant backgrounds: PIF protein levels in *phyB* mutants exceed wild-type (WT) levels, while phyB accumulates to high levels in *pif* mutants (Khanna *et al.*, 2007; Al-Sady *et al.*, 2008; Leivar *et al.*, 2008a; Jang *et al.*, 2010).

Recent work has linked certain PIFs to regulation of flowering time in *Arabidopsis*. *PIF4* is proposed to underlie a flowering time quantitative trait locus (Brock *et al.*, 2010). In addition, PIF4 modulates thermal induction of flowering by directly binding to and activating expression from the *FT* promoter (Kumar *et al.*, 2012). Warmer temperatures appear to stabilize PIF4 (Foreman *et al.*, 2011; Kumar *et al.*, 2012), and this may provide a mechanism by which PIF4 regulates *FT* in a temperature-dependent manner. The circadian clock controls *PIF4* and *PIF5* expression (Oda *et al.*, 2004; Nozue *et al.*, 2007; Thines and Harmon, 2010; Dixon *et al.*, 2011). A three-protein assembly known as the evening complex (EC) confers morning-phased expression to each gene (Nusinow *et al.*, 2011). The EC, which is composed of EARLY FLOWERING 3 (ELF3), LUX ARRHYTHMO (LUX), and EARLY FLOWERING 4 (ELF4), represses transcription of *PIF4* and *PIF5* by binding their promoters. As a consequence of clock and light regulation, PIF4 and PIF5 proteins accumulate in a short time window just prior to dawn (Nozue *et al.*, 2007; Yamashino *et al.*, 2013).

While other studies have investigated the effects of constant warm temperature on plant physiology, the present study investigated whether the regulatory networks that control flowering time distinguish between warm temperature cues perceived in the daytime and those present during night-time. The approach was to ask whether a window of enhanced sensitivity exists for thermal induction of flowering. *Arabidopsis* plants were evaluated for accelerated flowering brought on by combining warm temperature with either the light or dark period of a 24h diel photocycle. Plants flowered more quickly when exposed to warm nights (WN) compared with a moderate temperature control condition, and the WN condition was nearly as effective as constant warm (CW) temperature for induction of early flowering. Warm days (WD) also stimulated flowering, but less effectively than WN. Plants exposed to WN conditions exhibited strong up-regulation of *FT* expression that was absent from WD plants. Our findings implicate PIF4 and PIF5 in a signalling pathway that stimulates *FT* expression in a largely CO-independent manner. *pif4-2* and *pif5-3* single mutants had delayed flowering in any warm condition, and *pif4-2 pif5-3* double mutants showed delayed flowering in all conditions. Importantly, WN induction of *FT* was much reduced in the *pif4-2 pif5-3* background without a change in *CO* expression. Together, these findings demonstrate that PIF4 and PIF5 act together to match floral development to the light and temperature environment.

## Materials and methods

### Plant materials and growth conditions

All plants were in the Columbia-0 background. Seed for *pif4-2* (Leivar *et al.*, 2008*a*), *pif5-3* (Khanna *et al.*, 2007), and *pif4-2 pif5-3* (Nozue *et al.*, 2007) were a gift from Dr Peter Quail (Plant Gene Expression Center, Albany, CA, USA). *co-9* (Balasubramanian *et al.*, 2006) was obtained from the Arabidopsis Biological Resource Center at The Ohio State University. The PIF4-FLASH and PIF5-FLASH lines were a gift of Drs Joanne Chory and Ullas Pedmale (The Salk Institute, La Jolla, CA, USA). The FLASH tag consisted of a tandem combination of a c-Myc epitope (EQKLISEEDL), a 6×His-tag, and three copies of the FLAG epitope (DYKDDDDK). The *Cauliflower mosaic virus* 35S promoter drove constitutive expression of PIF4-FLASH and PIF5-FLASH. Construction of the PIF4-FLASH and PIF5-FLASH lines will be described elsewhere (U. Pedmalle and J. Chory, personal communication).

Surface-sterilized seeds were stratified for 3–5 d at 4 °C. At the first dark to light transition of day 1 [designated Zeitgeiber Time 0 (ZT0); typically 06:00 h], seeds in liquid MS (Murashige and Skoog) medium were placed in Percival growth chambers (Percival Scientific, www.percival-scientific.com), which were set to constant 22 °C and 12h light:12h darkness. Three hours later, the seeds were sown onto sterile filter paper (Whatman, www.whatman.com) in 100 mm×15mm square plates (BD Biosciences, www.bdbiosciences.com) containing 40ml of MS medium at pH 5.8 with 0.8% type I micropropagation agar (Cassion Laboratories, www.caissonlabs.com). Plants were transferred to new environmental conditions on day 4, after three complete photocycles. Lighting conditions for all experiments consisted of 12h of white light followed by 12h of darkness. Temperature conditions were constant 22 °C for control, 28 °C light periods (ZT0–ZT12) for WD, 28 °C dark periods (ZT12–ZT24) for WN, and constant 28 °C for CW. Temperature was confirmed with HOBO U10-003 data loggers (Onset, www.onsetcomp.com) placed in the chamber for at least 48h.

### Flowering time analysis

Seedlings were germinated as described above and then sown directly onto 4 inch plastic pots filled with wetted soil. Thereafter, plants were grown in Econair (Econair Technologies, Inc., www.biochambers.com) growth chambers set as described above. Pots were watered from overhead approximately every 3 d. The total number of rosette leaves was counted when the influorescence reached 1cm tall.

### Real-time PCR

Entire seedlings were harvested at the indicated times starting at ZT0 on day 14 of the experiment, placed in 1.5ml microcentrifuge tubes, and immediately frozen in liquid N_2_. A green LED light (PhotonLights, www.photonlight.com) was used for collection during dark periods. Samples were pulverized with 3.2mm stainless steel beads (Next Advance, Inc., www.nextadvance.com) with a MixerMill 301 (Retsch GmbH, www.retsch.com) in liquid N_2_. Real-time PCR (qPCR) was used for gene expression analysis. Total RNA was purified from ground tissue samples with Plant RNA reagent according to the manufacturer’ s recommendations (Invitrogen, www.invitrogen.com). Contaminating genomic DNA was removed with the TURBO DNA-free kit (Ambion, www.ambion.com). First-strand cDNA was prepared from 1 μg of DNase-treated RNA with the Maxima Universal First Strand cDNA Synthesis kit (Fermentas, www.fermentas.com) and diluted 1:5 prior to use. Transcript levels were determined with qPCR using a CFX96 Real-Time PCR Detection System (Bio-Rad, www.bio-rad.com) as described previously (Harmon *et al.*, 2008).

### Whole-cell extracts and western blot analysis

Entire PIF4-FLASH and PIF5-FLASH seedlings were grown in the indicated conditions and samples were harvested at ZT12 and ZT16 on day 14 of the experiment. After pulverizing the tissue as described above, whole-cell extracts were prepared by the hot extraction method described previously (Al-Sady *et al.*, 2006), except the protease inhibitor mix was the cOmplete, EDTA-free Protease Inhibitor Cocktail (Roche Applied Science, www.roche-applied-science.com). Proteins were separated on 10% SDS–polyacrylamide gels and western blotting was performed as described previously (Harmon *et al.*, 2008). Proteins with the FLASH epitope were detected with an equal mix of OctA-probe (D-8) and c-Myc (A-14) antisera (Santa Cruz Biotechnology, www.scbt.com) as primary antibodies, followed by goat anti-rabbit IgG–horseradish peroxidase (HRP) conjugate (Santa Cruz Biotechnology, www.scbt.com) as the secondary antibody.

## Results

### Warm nights and days promote flowering to produce unequal flowering times

**Fig. 1.**
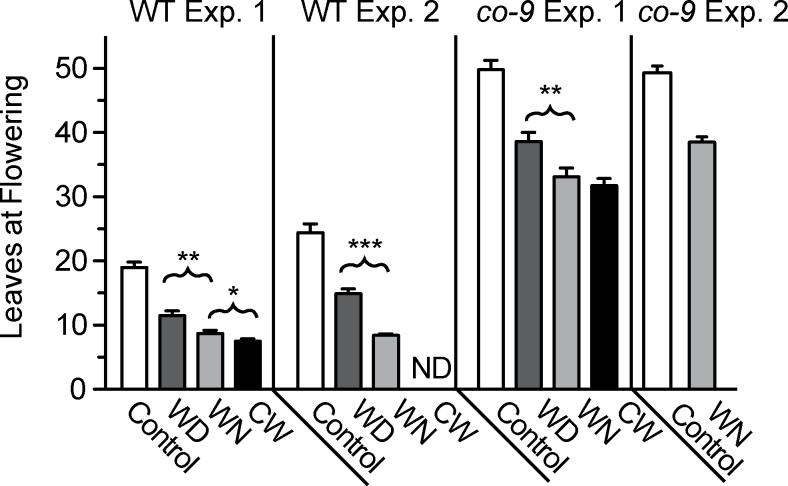
Warm temperature elicits different degrees of early flowering according to its timing relative to the photoperiod. Total leaf number of the wild type (WT) or *co-9* mutant grown in 12h of white light (50 μmol m^–2^ s^–1^), followed by 12h of darkness together with the indicated temperature condition. Conditions were: control (white bars), WD (dark grey bars), WN (light grey bars), and CW (black bars). Leaves at flowering included the rosette leaves produced when the influorescence reached 1cm. The results of two independent experiments are shown. The total number of WT plants for experiment 1 was 10 (control), 10 (WD), 18 (WN), and 16 (CW); for experiment 2, it was 25 (control), 32 (WD), and 84 (WN). The total number of *co-9* plants for experiment 1 was nine (control), eight (WN), 10 (WD), and nine (CW), and for experiment 2 was seven (control) and 10 (WN). Error bars are the standard error of the mean. Brackets above bars indicate *P*-values of <0.05 (*), <0.01 (**), and <0.001 (***) produced by an unpaired two-tailed *t*-test between the two populations indicated by the ends of the bracket.

The flowering time of *Arabidopsis* plants was measured in conditions where warm temperature (28 °C) was provided coincident with either daytime (WD) or night-time (WN), instead of continuously throughout the 24h cycle (CW). WT *Arabidopsis* plants experiencing any period of warm temperature flowered substantially earlier than those in the control condition of continuous 22 °C (Fig. 1); however, WD and WN treatments had unequal effects on flowering time. WN treatment induced more rapid flowering than did WD (Fig. 1). The effect of WN on flowering time was comparable with that observed for CW, except that the plants in CW flowered somewhat earlier than in WN. A second experiment confirmed that plants in WN conditions flowered earlier than plants exposed to WD (Fig. 1). When the light intensity was increased by 2.5-fold to 125 μmol m^–2^ s^–1^, the positive effect of WN on flowering time remained, as did the relative difference in behaviour between WN and WD (Supplementary Fig. S1 available at *JXB* online). These findings indicate that coincidence of warm temperature and night-time was a stronger stimulus for flowering than the combination of the same warm temperature and daytime.

### Warm temperature modifies the expression waveform of *FT* and *CO*

Warm temperature cues may have stimulated flowering through changes in the expression level or waveform (i.e. shape of the curve) of the floral integrators CO and FT. To test this possibility, *FT* and *CO* transcript levels were measured by qPCR in 2-week-old WT plants at 4h intervals over a total of 28h. Measurements began with the dark to light transition on the morning of day 14 at ZT0, and continued until ZT28, or 4h into the morning of day 15.

**Fig. 2.**
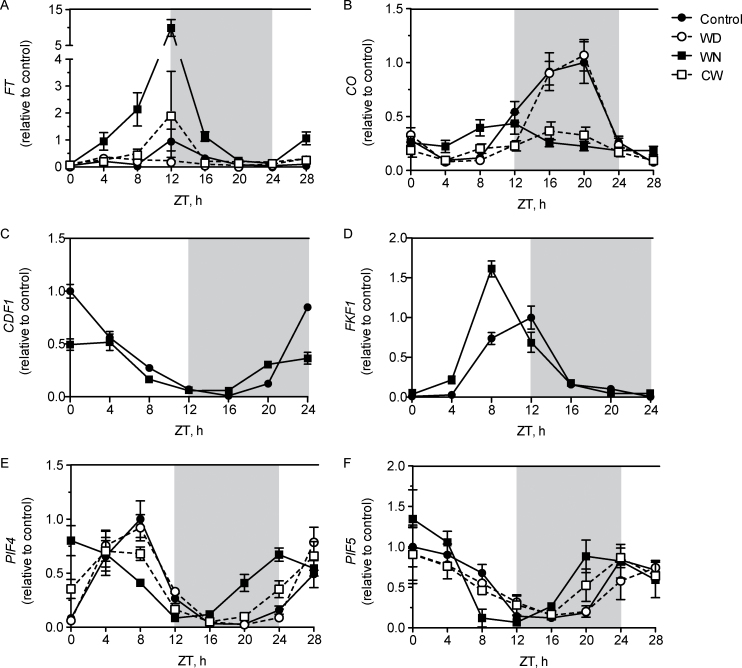
Warm treatment changes the expression pattern of genes governing flowering time and warm temperature responses. Expression of *FT* (A), *CO* (B), *CDF1* (C), *FKF1* (D), *PIF4* (E), and *PIF5* (F) in 2-week-old WT plants grown under control (filled circles, solid line), WD (open circles, dotted line), WN (filled squares, solid line), and CW (open squares, dotted line) conditions. Transcript levels were determined with qPCR and each time point was normalized to the time point from control conditions with the highest value. Each time point is the average of three biological replicates and the error bars are the standard error of the mean. The grey region denotes the ZT times in hours corresponding to the dark period.

Under control conditions, *FT* expression was rhythmic and reached peak levels in the evening at ZT12 (Fig. 2A), which is consistent with its established expression profile (Kobayashi *et al.*, 1999). Unexpectedly, *FT* expression in WD-grown plants lacked an obvious peak at ZT12 or at any other time during the time course (Fig. 2A), yet plants flowered earlier in this condition (Fig. 1). This observation indicated that earlier flowering in WD might not arise from elevated *FT* expression. However, it remained possible that WD could have affected *FT* expression in more mature plants closer to the time of flowering.

In contrast, *FT* transcripts in WN reached much higher levels than observed in plants from control conditions or WD, but expression continued to peak at ZT12 (Fig. 2A). The time over which *FT* expression occurred also increased: *FT* induction began immediately after dawn and rose throughout the day to culminate in the peak at ZT12. Plants grown in CW also showed higher *FT* expression, but the early rise was absent and the peak at ZT12 was substantially lower than in WN-grown plants (Fig. 2A).

Since CO is a direct transcriptional regulator of *FT* (Samach *et al.*, 2000), a plausible mechanism for *FT* up-regulation by warm temperature treatment was modification of *CO* expression. *CO* transcript levels in control plants showed the expected rhythmic waveform (Suarez-Lopez *et al.*, 2001), with *CO* expression rising in the light period between ZT8 and ZT12 and peak expression in the dark period at ZT16–ZT20 (Fig. 2B, filled circles). The *CO* accumulation in the light period was probably responsible for the sharp peak in *FT* expression at ZT12, since the leading shoulder of *CO* expression contributes to *FT* up-regulation (Suarez-Lopez *et al.*, 2001; Yanovsky and Kay, 2002; Imaizumi *et al.*, 2003; Valverde *et al.*, 2004).

Interestingly, rhythmic *CO* expression in WN-grown plants was advanced by 4h into the light period so that it began to rise earlier than in control plants at ZT4–ZT8 and reached peak levels between ZT8 and ZT12. *CO* levels fell after this time, unlike plants exposed to control or WD conditions. The waveforms of *GI*, *CDF1*, and *FKF1* expression were tested to examine the mechanism behind the phase advance for *CO*. At ZT8 to ZT12, where *CO* expression comes on in WN, the expression of *CDF1* and *GI* was largely unchanged (Fig. 2C; Supplementary Fig. S2 at *JXB* online). On the other hand, WN caused a 4h advance in *FKF1* expression so that the transcript reached a peak at ZT8 that was 2.5-fold higher than in the control condition at this time (Fig. 2D). This phase advance for *FKF1* matched the advance observed for *CO* (Fig. 2B); thus, the change in *FKF1* waveform in WN is the likely cause for the *CO* phase advance in this condition.

Exposure of plants to WD appeared to have an opposite effect on *CO* expression: *CO* expression began to rise in the dark after ZT12 and reached a peak of the same magnitude and phase as seen in plants under control conditions (Fig. 2B). The shift of *CO* expression into the dark part of the photoperiod correlated well with the low *FT* expression at ZT12 and throughout the remainder of the time course.

Finally, the amplitude of *CO* expression in CW conditions was reduced relative to both control and WD (Fig. 2B). In addition, lower relative expression for *CO* in CW compared to WN matched the reduced peak of *FT* expression in CW (Fig. 2A). Therefore, the WN condition appeared to be the only warm treatment that altered *CO* expression in a way that could explain the observed *FT* expression phenotype.

### Acceleration of flowering in WN occurs without CO activity

CO is not always required to promote *FT* expression in constant warm conditions (Blazquez *et al.*, 2003; Kumar *et al.*, 2012). To test whether CO was involved in earlier flowering in any of the warm temperature conditions, the flowering response of plants lacking CO function was evaluated in control, WD, WN, and CW conditions. The *co-9* mutant is a loss-of-function mutant caused by a T-DNA insertion in the *CO* gene (Balasubramanian *et al.*, 2006). As expected, *co-9* mutant plants exhibited substantially delayed flowering in all the temperature conditions (Fig. 1; Supplementary Fig. S1 at *JXB* online); however, *co-9* plants exposed to a period of warm temperature flowered earlier than in control conditions and exhibited the same relative response to all three conditions. Thus, the *co-9* plants maintained the capacity to accelerate flowering in response to warm cues. A nearly 3-fold induction of *FT* was apparent in *co-9* plants at ZT12, while WD had no positive effect on *FT* expression in the mutant background (Supplementary Fig. S3). This result shows that *CO* was not the only contributor to up-regulation of *FT* expression in WN. Therefore, CO is responsible for a large part of the response in WN, which probably underlies the differential flowering time between WD and WN, but a CO-independent mechanism is also involved.

### Warm nights change the phase of *PIF4* and *PIF5* expression

To understand the regulatory factors involved in WD- and WN-stimulated flowering, it was investigated whether these conditions modified the expression level or waveform of other genes known to control thermal-induced flowering. The *FVE*, *FCA*, *FLM*, and *FLC* genes have established roles in regulating flowering according to the temperature environment (Blazquez *et al.*, 2003; Balasubramanian *et al.*, 2006). None of these genes had notable differences in expression between control, WD, WN, and CW conditions (Supplementary Fig. S4 at *JXB* online).

*PIF4* and *PIF5* had expression waveforms in WN that were substantially different from those in any other environmental condition. *PIF4* and *PIF5*, which usually have distinct waveforms, adopted a similar early morning phase in WN (Fig. 2E, F). In all conditions other than WN, *PIF4* expression reached peak levels near ZT8, while in WN it coincided with ZT24; thus, this gene experienced an 8h phase advance only when the dark period was warm (Fig. 2E). Peak *PIF5* expression in WN was similarly advanced 4h earlier than in all other growth conditions (Fig. 2F). WN treatment did not promote higher expression of either transcript; instead, maximum *PIF4* and *PIF5* transcript levels remained close to the peak level observed in control plants. Since *PIF4* and *PIF5* are commonly regulated by the EC, the effect of WN on the expression waveforms for *LUX* and *ELF3* was investigated. Neither *LUX* nor *ELF3* was expressed in a manner consistent with the new phase for *PIF4* and *PIF5* (Supplementary Fig S2C, D at *JXB* online). The shift in *PIF4* and *PIF5* expression in response to WN was not observed for the *PIF* gene *LONG HYPOCOTYL IN FAR RED 1* (*HFR1*) (Fairchild *et al.*, 2000) (Supplementary Fig. S2B), which acts together with PIF4 and PIF5 in shade avoidance (Hornitschek *et al.*, 2009) and also contributes to warm temperature responses (Foreman *et al.*, 2011). The specific phase advance of *PIF4* and *PIF5* expression suggested a link between flowering promoted by WN and the activity of PIF4 and PIF5.

### PIF4 and PIF5 act both redundantly and additively to promote flowering

**Fig. 3.**
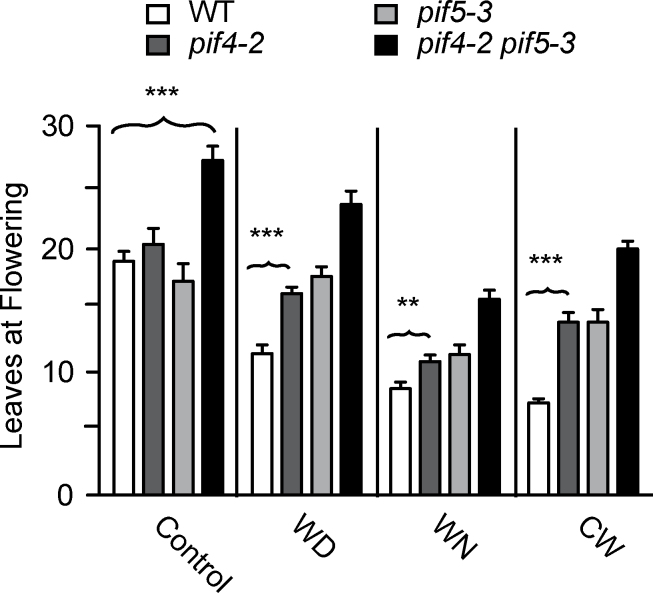
Loss of *PIF4* and *PIF5* attenuates the positive effects of warm temperature cues on flowering time. Flowering time of WT (white bars), *pif4-2* (dark grey bars), *pif5-3* (light grey bars), and *pif4-2 pif5-3* (black bars) plants. Growth conditions were as in Fig. 1 and leaves at flowering were determined by the same method. Each bar is the average of two biological replicates and error bars are the standard error of the mean. The total number of plants was: WT = 10 (control), 10 (WD), 18 (WN), 16 (CW); *pif4-2* = 10 (control), 10 (WD), 15 (WN), 15 (CW); *pif5-3* = 8 (control), 9 (WD), 16 (WN), 15 (CW); and *pif4-2 pif5-3* = 9 (control), 8 (WD), 16 (WN), 15 (CW). Brackets and symbols above bars represent a *P*-value of <0.01 (**) and <0.001 (***) produced by an unpaired two-tailed *t*-test between the indicated population and the WT grown under the same condition.

To assess whether PIF4 and PIF5 participate in stimulating flowering under warm temperature conditions, the flowering time of the single *pif4-2* and *pif5-3* mutants, as well as that of the *pif4-2 pif5-3* double mutant, was evaluated. Under control conditions, *pif4-2* and *pif5-3* plants flowered with an indistinguishable number of leaves compared with the WT (Fig. 3); therefore, neither of these genes alone was required to promote flowering at constant 22 °C. However, the *pif4-2 pif5-3* double mutant flowered significantly later than the WT in control conditions, generating on average eight more leaves than the WT (Fig. 3). This observation showed that *PIF4* and *PIF5* play redundant roles in flowering time at continuous 22 °C.

Previous work showed delayed flowering for the *pif4-101* allele in short day photoperiods at constant 28 °C (Kumar *et al.*, 2012). Similarly, the *pif4-2* allele had significantly delayed flowering in 12:12h photoperiods when warm temperature cues were present in the context of WD, WN, and CW (Fig. 3). *pif5-3* plants exposed to 28 °C had the same general trend for flowering time as the *pif4-2* mutant (Fig. 3). In either WN or WD, the single *pif4-2* and *pif5-3* mutants had ~1.5-fold more leaves at flowering compared with the WT, while each mutant made nearly twice the number of leaves at flowering in CW. Thus, *PIF4* and *PIF5* were both required for full acceleration of flowering time in response to warm temperature, regardless of the timing for the temperature cue.

The *pif4-2 pif5-3* double mutant displayed considerable delays in flowering time under all warm conditions that were stronger than in continuous 22 °C (Fig. 3). In general, double mutant plants exposed to warm cues flowered with more than twice the number of leaves than the WT. However, the severity of the phenotype depended on the type of temperature treatment. The delayed flowering phenotype of *pif4-2 pif5-3* plants was greatest in the CW condition, where the plants made nearly three times more leaves than WT plants under the same conditions. These findings show that PIF4 and PIF5 are redundantly required for the signalling pathways that control thermal induction of flowering.

### Loss of both *PIF4* and *PIF5* reduces *FT* expression in WN without a substantial change in *CO* expression

**Fig. 4.**
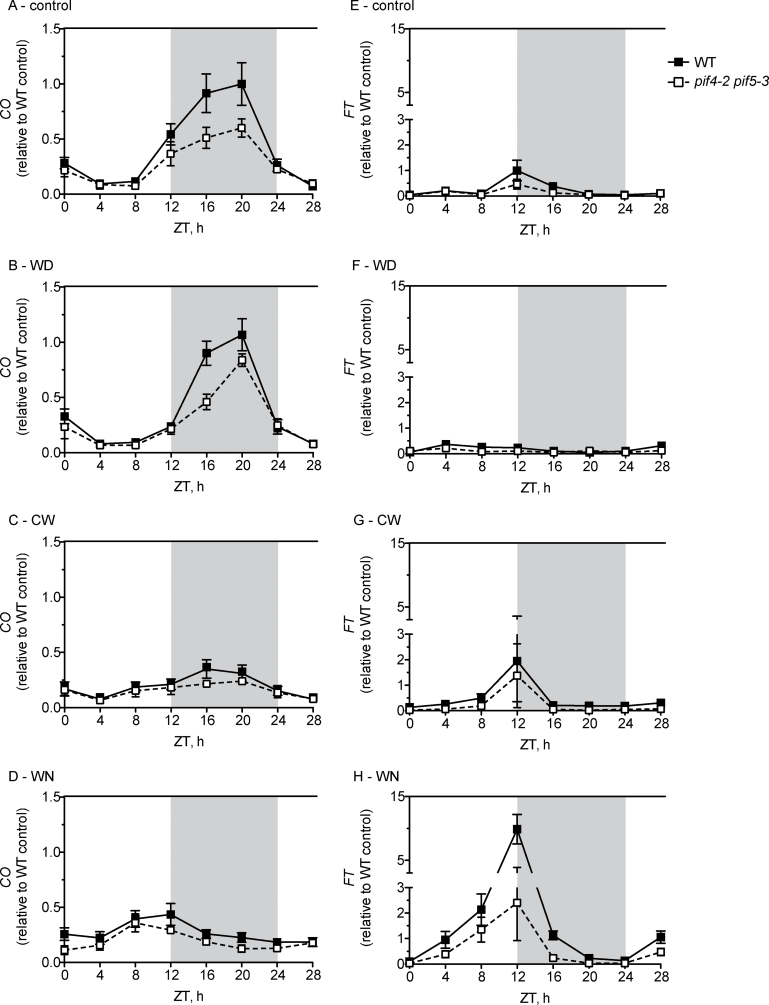
The *pif4-2 pif5-3* double mutant substantially reduces WN-induced *FT* expression without major changes in the *CO* expression waveform. Expression of *FT* (A–D) and *CO* (E–H) in 2-week-old WT (filled squares, solid line) and *pif4-2 pif5-3* mutant (open squares, dotted line) plants grown under control (A, E), WD (B, F), CW (C, G), and WN (D, H). *pif4-2 pif5-3* sampling was done at the same time as that for the WT in Fig. 2. The WT data from Fig. 2 are re-plotted here for comparison. Transcript levels were determined with qPCR and each time point was normalized to the highest value time point from the WT under the control conditions in Fig. 2. Each time point is the average of three biological replicates and error bars are the standard error of the mean. The grey region denotes the ZT times in hours corresponding to the dark period.

To understand how PIF4 and PIF5 promote induction of flowering, the effect of the double *pif4-2 pif5-3* mutant on the expression waveforms of *CO* and *FT* in the four environmental conditions was investigated. *CO* expression in the *pif4-2 pif5-3* mutant was largely the same as that of the WT during the light part of the photoperiod regardless of the condition (Fig. 4A–D). In the control condition, the double mutant had lower *CO* expression in the dark period beginning at ZT12 and continuing until dawn at ZT24 (Fig. 4A). A similar reduction for *CO* was also apparent in WD-grown mutant plants, but the magnitude of change was lower (Fig. 4B). The *CO* waveform had slightly lower amplitude in *pif4-2 pif5-3* for both WN and CW, but the timing of the peak was similar to that of WT plants. Since CO protein is turned over in the dark (Valverde *et al.*, 2004), the observed lower *CO* expression under control and WD conditions in this mutant background seemed unlikely to translate into lower CO protein. Therefore, no strong effect on *CO* expression was observed in the double mutant that agreed with the observed flowering phenotypes.

Except for in WN-grown plants, the *FT* waveform in *pif4-2 pif5-3* correlated well with that of *CO*. *FT* expression in control conditions was only slightly lower than in the WT, possibly due to the small difference in *CO* expression between the two genotypes at ZT12 (Fig. 4E). In WD conditions, *FT* expression was lower than control in the WT and *pif4-2 pif5-3* (Fig. 4F), which is in agreement with peak *CO* waveforms coinciding with the dark part of the photoperiod (Fig. 4B). In fact, strong *FT* up-regulation was absent from plants of either genotype grown in WD, yet both WT and *pif4-2 pif5-3* plants flowered earlier in this condition (Fig. 3). This observation reinforces the notion that an *FT*-independent mechanism was responsible for early flowering in WD. *FT* expression in the WT and *pif4-2 pif5-3* was similar under CW and was slightly elevated compared with control conditions, but lower than in WN (Fig. 4H).

In stark contrast, *pif4-2 pif5-3* plants had much lower *FT* expression when grown in WN: peak *FT* transcript levels reached only 20% of that in WT plants in the same condition (Fig. 4G). However, the lower *FT* expression in the *pif4-2 pif5-3* background did not correlate well with the marginal reduction or phase advance of *CO* expression. It is noteworthy that *FT* expression was lower at ZT4 and ZT8 in the *pif4-2 pif5-3* background, which is a time when CO was unlikely to promote expression. Thus, PIF4 and PIF5 were most important for *FT* expression in WN conditions and their action did not necessarily rely on modification of *CO* expression. Taken together, these data point to PIF4 and PIF5 promoting *FT* expression through a CO-independent mechanism.

### PIF4 and PIF5 protein reach comparable levels in control and WN conditions

A key mechanism to control PIF4 and PIF5 activity is post-transcriptional regulation of protein levels (Shen *et al.*, 2007; Lorrain *et al.*, 2008). In general, PIF proteins are stable in the dark and are rapidly degraded in the light by virtue of their interaction with phyB (Al-Sady *et al.*, 2006; Leivar and Quail, 2011). Continuous 27 °C has been reported to stabilize PIF4 somewhat during the early morning light period (Kumar *et al.*, 2012). In light of the fact that PIF4 directly binds to the *FT* promoter to regulate expression (Kumar *et al.*, 2012; Yamashino *et al.*, 2013), a potential explanation for the positive effects of WN on *FT* expression was that this warm condition results in higher levels of PIF4, PIF5, or both proteins. To test this possibility, the levels of PIF4 and PIF5 protein were examined in control and WN-grown plants at ZT12 and ZT16, which is when *FT* expression was greatest. Transgenic lines with constitutive expression of FLASH epitope-tagged PIF4 or PIF5 were used to detect these proteins. In either control or WN conditions, both PIF4-FLASH and PIF5-FLASH were detected at each of these evening time points and the protein levels were comparable across the conditions within a given experimental replicate (Supplementary Fig. S4 at *JXB* online). Thus, the WN condition did not favour accumulation of more PIF4 or PIF5 protein. Because PIF4 and PIF5 were constitutively expressed in these lines, the unvarying protein levels between control and WN plants indicated that post-transcriptional regulation of PIF4 and PIF5 was not changed substantially by WN treatment.

## Discussion

Exposure of *A. thaliana* to warmer temperatures causes early flowering (Blazquez *et al.*, 2003; Halliday *et al.*, 2003). The signalling pathways that sense and respond to warm temperature cues are incompletely understood. Most previous studies have focused on the effects of continuous warm temperature, whereas the present study tested for selective sensitivity to warm cues at defined parts of a photoperiod. This investigation shows that, like CW conditions, WN and WD conditions accelerate flowering, but the effectiveness of each was unequal: plants in WN flowered earlier than those in WD (Fig. 1; Supplementary Fig. S1 at *JXB* online) and each condition induced distinct gene expression patterns for the flowering time genes *CO* and *FT* (Fig. 2). Additionally, *PIF4* and *PIF5* were required for warm-induced flowering in all conditions, but WN alone changed the expression pattern of these genes. These results show that PIF4 and PIF5 are critical transcription factors for thermal-induced flowering in *Arabidopsis*.

### The timing of warm temperature has differential effects on FT and *CO*

Expression of *CO* and *FT* elicited by WN and WD showed a flexible warm temperature response that, on one hand, acts through the circadian clock to fine-tune *FT* expression, but also promotes flowering without modulating *FT* expression. In WN, higher *FT* expression throughout the day culminated in 10-fold higher transcript levels at dusk relative to the control at the same time (Fig. 2A). This was the strongest *FT* expression change elicited by any of the three warm conditions. Simultaneously, peak rhythmic expression of *CO* remained lower than the control but occurred much earlier during the light part of the photoperiod (Fig. 2B). The phase advance of *CO* readily explained *FT* up-regulation in the WN condition, as it likely increased active CO abundance. A less substantial phase advance that shifts *CO* expression into the light period underlies the early flowering phenotype of the *toc1-1* circadian clock mutant (Yanovsky and Kay, 2002). An advance in *FKF1* expression to earlier in the light period was also apparent in WN, and this matched the timing of earlier *CO* expression, which provides a plausible mechanism for up-regulation of *CO* at this time. Thus, early flowering in WN appeared to be promoted in part by CO-mediated up-regulation of *FT* expression.

WN conditions not only advanced the phase of *CO* expression, but also reduced the overall amplitude of the *CO* expression waveform (Fig. 2B). A similar reduction in *CO* expression was also evident in CW, but peak *CO* expression was timed to the middle of the dark period. The reduction in *CO* amplitude observed in WN and CW was analogous to the dampening of night-time *CO* expression observed in plants grown at constant 23 °C compared with 16 °C (Blazquez *et al.*, 2003).

### FT- and CO-independent flowering stimulated by warm cues

Flowering in WD appeared to rely less on the *CO*/*FT* module. WD conditions largely shifted *CO* expression into the dark (Fig. 2). Predictably, *FT* showed no induction in WD and was even slightly repressed relative to control samples. It is likely that reduced *FT* expression in WD was a consequence of the altered phasing of *CO* expression. Although *FT* was poorly expressed in WD, plants grown in this condition flowered much earlier than those in control conditions (Fig. 1), which indicates the action of a temperature-sensitive *FT*-independent flowering pathway. At least two *FT*-independent flowering pathways are known (Wilson *et al.*, 1992; Blazquez and Weigel, 2000; Reeves and Coupland, 2001; Balasubramanian *et al.*, 2006; Han *et al.*, 2008), and it will be interesting to test whether these contribute to earlier flowering in WD conditions.

A *co* mutant flowered early in WN, WD, and CW, showing that warm cues can induce flowering without *CO* (Fig. 1), which is consistent with previous work (Balasubramanian *et al.*, 2006). Furthermore, the persistence of a differential flowering response to WN and WD in *co-9* plants indicates that other factors contribute to the time of day warm temperature effects observed here. The significant flowering delay of *co-9* plants under all conditions, however, made it difficult to establish the relative contribution of each pathway to the WD and WN effects. FT acts in thermal induction of flowering by integrating signals from multiple pathways (Blazquez *et al.*, 2003; Balasubramanian *et al.*, 2006). FVE and FCA participate in *FT* induction at warm temperatures without the action of CO (Blazquez *et al.*, 2003). Furthermore, FLC is a repressor of flowering and FLM modulates thermal sensitivity (Balasubramanian *et al.*, 2006). Significant changes in expression of *FLC*, *FLM*, *FVE*, or *FCA* did not occur in WN during the part of the photoperiod where *FT* was induced (Supplementary Fig. S4 at *JXB* online). However, it is possible that post-transcriptional effects modify the function of FVE, FCA, FLC, or FLM to trigger early flowering in WN conditions.

### PIF4 and PIF5 regulate flowering time

Expression analysis led to the investigation of the contribution of PIF4 and PIF5 to the time of day-specific effects of warm temperature on flowering time. Peak expression of *PIF4* and *PIF5* in WN occurred just before dawn, which was significantly earlier than seen in any other condition (Fig. 2E, F). Since continuous warm temperatures generally shorten the clock period in *Arabidopsis*, a shorter period could be the cause of the phase advance; however, this possibility seems unlikely given that the *Arabidopsis* Columbia-0 accession employed here lengthens the period at warm temperatures near 28 °C (Edwards *et al.*, 2005, 2006).

The closely matched waveforms of *PIF4* and *PIF5* in WN indicate a common mechanism for advancing the phase of these genes. *PIF4* and *PIF5* expression is similarly phase advanced in plants at constant 28 °C and long-day photoperiods (Nomoto *et al.*, 2012*a*, *b*); however, this long day-specific effect is an improbable explanation, since the phase advance seen here was confined to WN and not generally seen in any warm condition. Interestingly, *PIF4* and *PIF5* expression in an *elf3* mutant shifts to the late night under diurnal conditions at 22 °C in a manner similar to that in WN (Nusinow *et al.*, 2011). However, *ELF3* and *LUX* expression in WN was not changed in a way that was consistent with the new *PIF4* and *PIF5* waveforms (Supplementary Fig. S2C, D at *JXB* online). A strong possibility that remains is that WN conditions modify the activity or formation of the EC complex and this induces the change in *PIF4* and *PIF5* expression. Precisely how WN conditions cause an advance phase in PIF4 and PIF5 expression remains to be determined.

*PIF4* and *PIF5* are needed to promote flowering, particularly in response to warm cues occurring throughout the photoperiod. Both the *pif4-2* and *pif5-3* alleles flowered normally under the control condition, but the *pif4-2 pif5-3* double mutant showed considerably delayed flowering in this condition. Thus, PIF4 and PIF5 are redundantly required for flowering at 22 °C. On the other hand, PIF4 and PIF5 appeared to contribute additively to flowering time in the presence of warm temperature cues. Individually, *pif4-2* and *pif5-3* showed delayed flowering under any of the three warm conditions tested here. The magnitude of the flowering delay for each single mutant was nearly equivalent in both WD and WN (Fig. 3). The delay in flowering observed for the *pif4-2* mutant is like that previously reported for the *pif4-101* allele at constant 28 °C (Kumar *et al.*, 2012). The flowering time delay of the *pif4-2 pif5-3* double mutant was about twice that of the single mutants. It is interesting to note that the greatest flowering delay in the double mutant background relative to WT was in CW conditions, yet the *pif4-2 pif5-3* plants flowered earliest in WN conditions, not in CW conditions like WT and the *co-9* mutant. One interpretation of this observation is that PIF4 and PIF5 have multiple roles in flowering, and in certain roles their action is sensitive to time of day. In this model, the WD- and WN-specific effects are negated when night and day warm cues are experienced at the same time as in CW.

Previous work demonstrated that PIF4 directly activates *FT* expression (Kumar *et al.*, 2012). The results here indicate that PIF5 may play a similar role in transcriptional regulation of *FT*. A second, but not mutually exclusive, role for these PIFs in flowering may be to modify phyB photoreceptor abundance. PIF4 and PIF5 physically interact with phyB, which results in the degradation of both the PIF and phyB proteins (Khanna *et al.*, 2007; Shen *et al.*, 2007; Leivar *et al.*, 2008*a*; Jang *et al.*, 2010). Correspondingly, phyB accumulates to higher levels in *pif4* and *pif5* mutants. Since phyB represses CO activity to delay flowering time (Guo *et al.*, 1998; Valverde *et al.*, 2004), the delayed flowering observed in the *pif* mutant backgrounds could stem from more abundant phyB. Additionally, phyB accumulation is inversely proportional to ambient temperature, so that less of the photoreceptor is present at warm temperatures (Foreman *et al.*, 2011). Consequently, PIF4 protein is more stable and achieves higher levels at constant 28 °C. However, PIF4 and PIF5 accumulation was not changed by WN conditions, at least in a constitutively expressed context (Supplementary Fig. S5 at *JXB* online). Halliday and colleagues found that phyB represses *FT* at 22 °C, and not at 16 °C, but at the same time had little effect on *CO* expression at both temperatures (Halliday *et al.*, 2003). This finding led to the conclusion that a phyB pathway converges on *FT* only at warmer temperatures, and its action is independent of CO and the photoperiod pathway. Clearly, additional work is needed to assess whether the PIF-dependent WN response observed here and the phyB pathway active at 22 °C represent a common mechanism.

The requirement for both PIF4 and PIF5 activity to achieve maximal *FT* induction under WN presents an interesting conundrum of how the dawn-phased and light-labile PIF proteins exert a meaningful effect on evening-phased *FT*. A partial explanation is provided by the observation that continuous 27 °C slightly stabilizes PIF4 up to 4h into the light period of the morning (Kumar *et al.*, 2012). The phase shift of *PIF4* and *PIF5* expression into the dark period caused by WN may create a large supply of protein that persists well into the day. Alternatively, association of PIF4 and PIF5 with the *FT* promoter in the pre-dawn hours may help to potentiate *FT* expression later in the day. Consistent with either proposal, *FT* transcript rises much earlier in the day in WN conditions and this was attenuated in the *pif4-2 pif5-3* background. Although the WN-stimulated early rise in *FT* expression is somewhat retained in the double mutant, it is possible that other PIF family transcription factors partially substitute for PIF4 and PIF5, as is the case in seedling photomorphogenesis (Leivar *et al.*, 2008*a*, *b*).

### Conclusion

The discrete WD and WN conditions imposed here were not meant to represent real-world conditions, but instead were tools to probe for the existence of time of day-specific warm temperature response pathways that promote flowering in *Arabidopsis* plants. The findings indicate that warm temperature elicits earlier flowering through several signalling pathways, which to varying degrees rely on the activity of *CO*, *FT*, *PIF4*, and *PIF5*. The precise combination of pathways depends on the time of the temperature cue. Of particular note was the much earlier flowering caused by the WN treatment, which raises the question of why *Arabidopsis* plants are attuned to the coincidence of night and warm temperature cues? It makes teleological sense for *Arabidopsis* to undergo more rapid flowering when ambient temperatures rise, since warm conditions can be indicative of oncoming warmer seasons or a portent of potential stresses such as heat and drought. Considering that daytime is typically warmer than night-time in natural conditions, it is reasonable to propose that the sensing system evolved to use the combination of elevated temperature and darkness as an indicator of potentially severe and/or long-term environmental changes that deviate from the past ‘normal’ conditions.

## Supplementary data

Supplementary data are available at *JXB* online.

Table S1. Primers used for qPCR.

Figure S1. WN conditions elicit early flowering in WT and *co-9* plants under high intensity white light.

Figure S2. WN causes limited changes to the expression waveform of *GI*, *HFR1*, *LUX*, and *ELF3*.

Figure S3. *co-9* mutant plants retain the capacity to induce *FT* expression in WN and CW conditions.

Figure S4. WN conditions do not substantially change evening expression of *FVE*, *FCA*, *FLM*, and *FLC*.

Figure S5. PIF4 and PIF5 accumulation is not substantially different between WN and control conditions.

Supplementary Data
