# Supplementary material for: The time of day effects of warm temperature on flowering time involve PIF4 and PIF5
Source: J Exp Bot. 2014 Feb 18;65(4):1141–51. doi: 10.1093/jxb/ert487 (PMC3935576; doi:10.1093/jxb/ert487)
Supplement: Supplementary Data [file supp_65_4_1141__index.html]

The time of day effects of warm temperature on flowering time involve PIF4 and PIF5 — Supplementary Data 

# The time of day effects of warm temperature on flowering time involve PIF4 and PIF5

## Supplementary Data

Data files

**Files in this Data Supplement:**

- Supplementary Data - Supplementary Data
