# Supplementary material for: The time of day effects of warm temperature on flowering time involve PIF4 and PIF5
Source: J Exp Bot. 2014 Feb 18;65(4):1141–51. doi: 10.1093/jxb/ert487 (PMC3935576; doi:10.1093/jxb/ert487)
Supplement: Supplementary Data [file supp_ert487_jexbot107797_file001.pdf]

The time of day effects of warm temperature on flowering time involve PIF4 and PIF5

Bryan C. Thines, Youngwon Youn, Maritza I. Duarte, and Frank G. Harmon

Supplementary Material

Table S1. Primers used for qPCR

Primer	Sequence (5’ to 3’)
CDF1-F	GCTGATGAAGAAGAAGAAAAGAACC
CDF1-R	GCTGTTGCATCTTGGACATGG
CO-F	AGCTGTGATGCTCAAGTTCACTCT
CO-R	GCAGACCCGGACACGTTTAT
ELF3-F	AGGCAAGGGAGCACAGGAA
ELF3-R	GCCGAAAGGACTTGCTACCA
FCA-F	TGTTCGAACGAGAGCAACAG
FCA-R	AACGGCTGTAATTGGGTCTG
FKF1-F	ACTCCGCCTTCGTTCATTGT
FKF1-R	AGGAATCGACAGTTACGACCA
FLC-F	CGCATCCGTCGCTCTTCT
FLC-R	CAAGGATCTTGACCAGGTTATCG
FLM-F	GCCAGATGGGAAAGAATACG
FLM-R	AGGTGAAAACTCAGCCGTTG
FVE-F	ACTGGGCACCAAGATAATGC
FVE-R	GTCCCAATCGTTGTGATGTG
FT-F	AAGGCCTTCTCAGGTTCAAA
FT-R	ATCAGTCACCAACCAATGGA
GI-F	ATGGTGTAGTGGTGTAATGGGTAAATAT
GI-R	CAGATCCTCGAGAAGCAATGG
HFR1-F	TGGAATGCACAACCACATGC
HFR1-R	GCCAATTTAACGCCGGAATT
IPP2-F	GTATGAGTTGCTTCTCCAGCAAAG
IPP2-R	GAGGATGGCTGCAACAAGTGT
LUX-F	TAACGTGGAGGAGGAAGATCGA
LUX-R	TCCATCACCGTTTGATGTCTTT
PIF4-F	CCCATCACAGAACGATCTCGAT
PIF4-R	AGGAGCCACCTGATGAGGAACT
PIF5-F	AATTCCCGGTTATGAACCGGT
PIF5-R	TACCTAGCGAGCTGCTCCGATA

Supplementary Data Legends

Figure S1. WN conditions elicit early flowering in WT and *co-9* plants under high intensity white light. Total leaf number of plants grown in 12 hours of white light, at a fluence rate of 125 µmole m^{-2} s^{-2}, followed by 12 hours of darkness under control (white bars), WD (dark grey bars), and WN (light grey bars). Total number of wild type (WT) plants assayed was 27 (control), 24 (WN), and 24 (WD). The total number of co-9 plants assayed was 16 (control) and 10 (WN). Total leaf number included the cauline and rosette leaves produced when the influorescence reached 1 cm. The results of two independent experiments are shown for WT and one experiment for *co-9*. Error bars are standard error of the mean. Brackets above bars indicate p-values of <0.05 (*) and <0.001 (***) produced by an unpaired two-tailed t-test between the two populations indicated by the ends of the bracket.

Figure S2. WN causes limited changes to the expression waveform of *GI*, *HFR1*, *LUX*, and *ELF3*. Expression of *GI* (A), *HFR1* (B), *LUX* (C), and *ELF3* (D) in two week-old WT plants grown under control (closed circles) or WN (closed squares) conditions. Transcript levels were determined with qPCR and each time point was normalized to the time point from control conditions with the highest value. Each time point is the average of three independent biological replicates and error bars are standard error of the mean. The grey region denotes the ZT times in hours (h) corresponding to the dark period.

Figure S3. *co-9* mutant plants retain the capacity to induce FT expression in WN and CW conditions. Expression of *FT* in two week-old WT plants at ZT12 grown under conditions of control (white bar), WD (dark grey bar), WN (light grey bar), and CW (black bar). Transcript levels were determined with qPCR and each time point was normalized to the ZT12 time point from control conditions in Fig. 1. Each time point is the average of three biological replicates and error bars are standard error of the mean.

Figure S4. WN conditions do not substantially change evening expression of *FVE, FCA, FLM,* and *FLC* . Expression of *FVE* (A), *FCA* (B), *FLM* (C), and *FLC* (D) in two week-old WT plants grown under control (white bars) or WN (light grey bars) at the indicated ZT times. Transcript levels were determined with qPCR and each time point was normalized to the ZT8 sample from control conditions. Each time point is the average of three independent biological replicates and error bars are standard error of the mean.

Figure S5. PIF4 and PIF5 accumulation is not substantially different between WN and control conditions.
Levels of PIF4-FLASH (A) and PIF5-FLASH (B) protein in two week-old plants that grown in either control or WN conditions. Samples were collected at either ZT12 or ZT16. Lanes labeled “no tag” were loaded with extracts from nontransgenic plants in control conditions collected at ZT12. The “no tag” lane is from the same membrane. Intervening lanes were removed for clarity in panel B, which is indicated by the white bar. Bands corresponding to PIF4-FLASH and PIF5-FLASH are labeled, as is the band used as loading control (LC). The LC was a non-specific band from the same membrane that was recognized by the primary antibodies. Proteins having the FLASH epitope were detected with an equal mix of OctA-probe and c-Myc antisera. Shown are results from two independent biological replicates. Panels C) and D) demonstrate that the PIF4-FLASH (P4) and PIF5-FLASH (P5) proteins are functional. When grown in constant white light conditions combined with either continuous 22°C or 28°C, six day-old seedlings harboring either PIF4-FLASH (grey bars) or PIF5-FLASH (black bars) transgenic construct have significantly elongated hypocotyls compared to wild type (WT; white bars) seedlings. Error bars in D) are standard deviation of populations of 16 seedlings and data is from one of two experiments with comparable results.

Figure S1

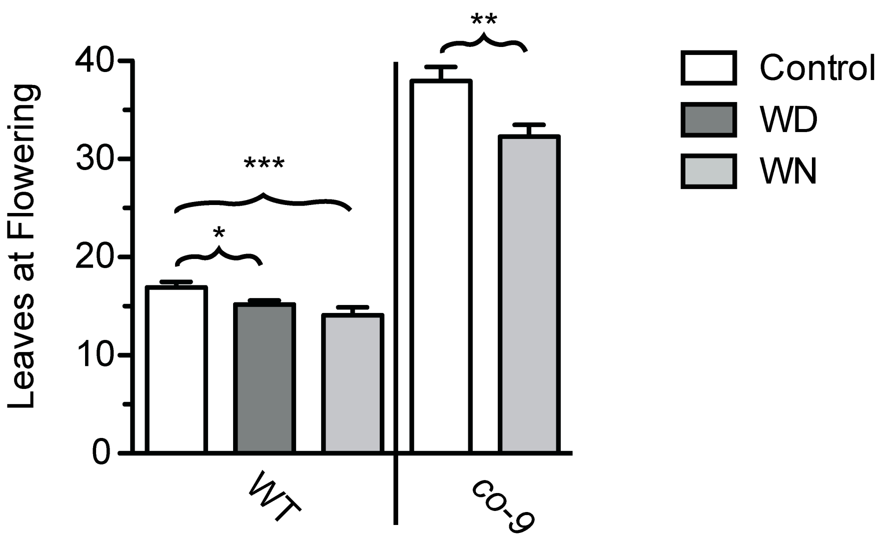

Figure S2

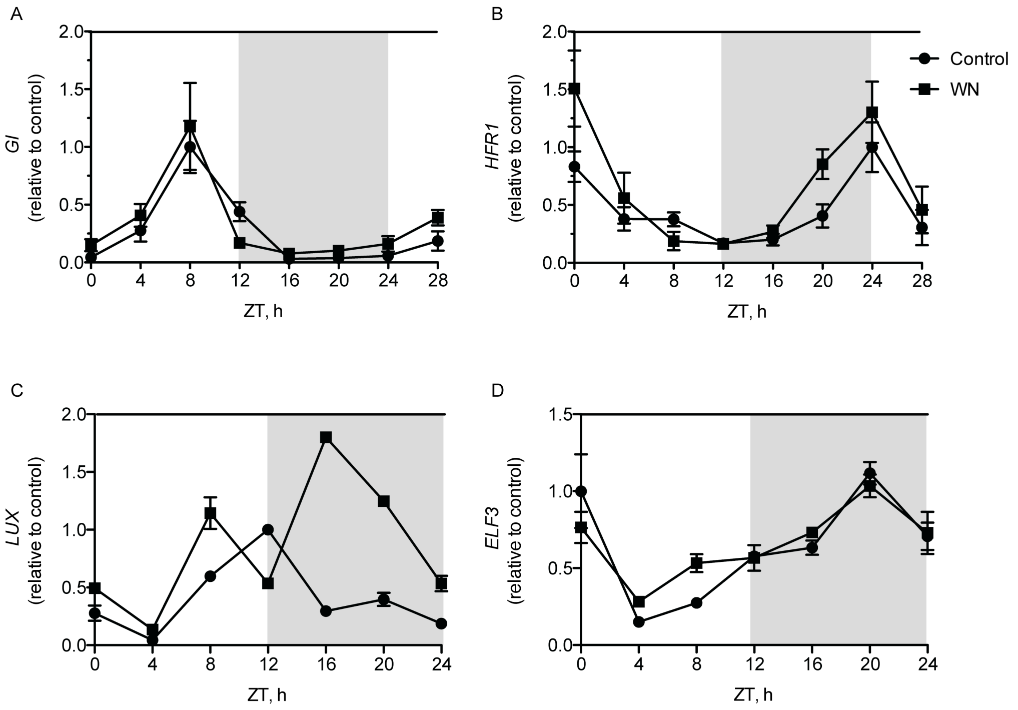
A
B
C
D
GI
(relative to control)
HFR1
(relative to control)
LUX
(relative to control)
ELF3
(relative to control)
ZT, h
Control
WN
2.0
1.5
1.0
0.5
0.0
0
4
8
12
16
20
24
28

Figure S3

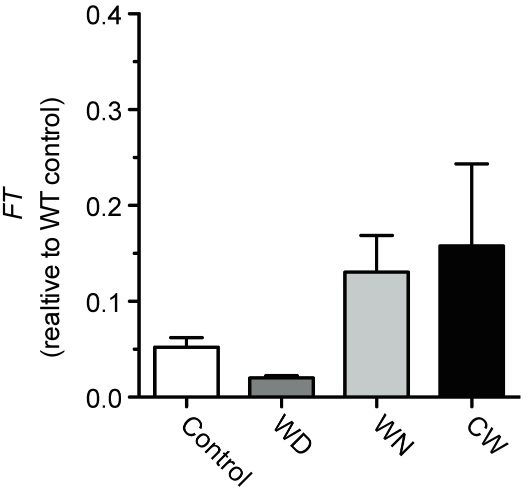

Figure S4

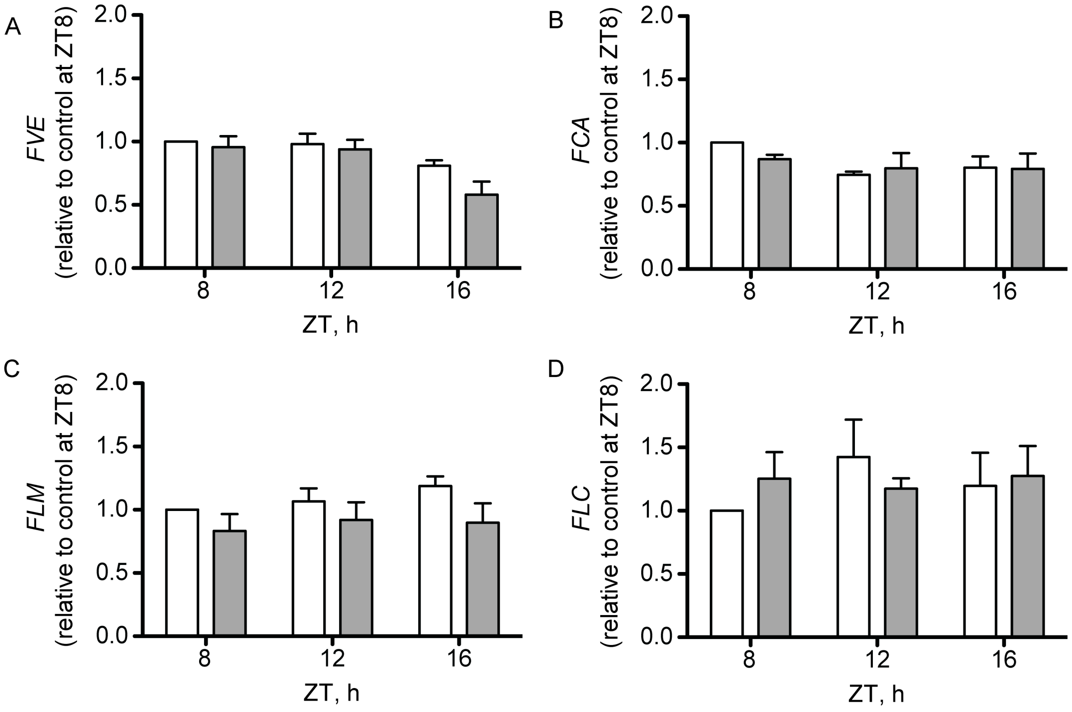

A
FVE
(relative to control at ZT8)
ZT, h
B
FCA
(relative to control at ZT8)
ZT, h
C
FLM
(relative to control at ZT8)
ZT, h
D
FLC
(relative to control at ZT8)
ZT, h

Figure S5

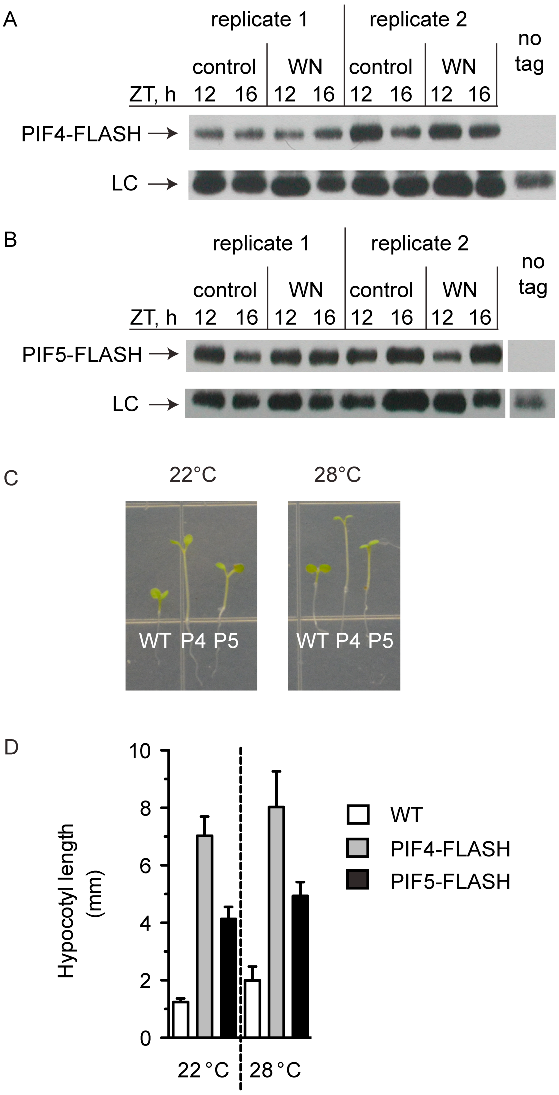
A
replicate 1
replicate 2
control
WN
control
WN
no tag
ZT, h 12 16 12 16 12 16 12 16
PIF4-FLASH
LC
B
replicate 1
replicate 2
control
WN
control
WN
no tag
ZT, h 12 16 12 16 12 16 12 16
PIF5-FLASH
LC
C
22°C
28°C
WT P4 P5
WT P4 P5
D
Hypocotyl length (mm)
10
8
6
4
2
0
22 °C
28 °C
WT
PIF4-FLASH
PIF5-FLASH
